# Supplementary material for: HIVprotI: an integrated web based platform for prediction and design of HIV proteins inhibitors
Source: J Cheminform. 2018 Mar 9;10:12. doi: 10.1186/s13321-018-0266-y (PMC5845081; doi:10.1186/s13321-018-0266-y)
Supplement: Supplementary file 2 — Additional file 2. Source code of HIVProtI web server. [file 13321_2018_266_MOESM2_ESM.zip › HIVprotI_Source-code/hivprotI/weka-3-6-6/doc/weka/core/UnassignedClassException.html]

UnassignedClassException


---


|  |  |  |
| --- | --- | --- |
| **Overview** \| **Package** \| **Class** \| **Tree** \| **Deprecated** \| **Index** \| **Help** \| **Weka's home** | |  |
| **PREV CLASS**   **NEXT CLASS** | **FRAMES**    **NO FRAMES**     **All Classes** |  |
| SUMMARY: NESTED \| FIELD \| CONSTR \| METHOD | DETAIL: FIELD \| CONSTR \| METHOD |  |


---


## weka.core Class UnassignedClassException

```
java.lang.Object
  java.lang.Throwable
      java.lang.Exception
          java.lang.RuntimeException
              weka.core.UnassignedClassException
```

**All Implemented Interfaces:**: java.io.Serializable

---

``` public class UnassignedClassException extends java.lang.RuntimeException ```

Exception that is raised when trying to use some data that has no
class assigned to it, but a class is needed to perform the operation.

**Version:**
:   $Revision: 1.5 $

**Author:**
:   Richard Kirkby (rkirkby@cs.waikato.ac.nz)

**See Also:**: Serialized Form

---

**Constructor Summary**

`UnassignedClassException()`
  
          Creates a new UnassignedClassException with no message.

`UnassignedClassException(java.lang.String message)`
  
          Creates a new UnassignedClassException.


**Method Summary**

**Methods inherited from class java.lang.Throwable**

`fillInStackTrace, getCause, getLocalizedMessage, getMessage, getStackTrace, initCause, printStackTrace, printStackTrace, printStackTrace, setStackTrace, toString`

**Methods inherited from class java.lang.Object**

`equals, getClass, hashCode, notify, notifyAll, wait, wait, wait`

**Constructor Detail**

### UnassignedClassException

```
public UnassignedClassException()
```

:   Creates a new UnassignedClassException with no message.

---


### UnassignedClassException

```
public UnassignedClassException(java.lang.String message)
```

:   Creates a new UnassignedClassException.

    **Parameters:**: `message` - the reason for raising an exception.


---


|  |  |  |
| --- | --- | --- |
| **Overview** \| **Package** \| **Class** \| **Tree** \| **Deprecated** \| **Index** \| **Help** \| **Weka's home** | |  |
| **PREV CLASS**   **NEXT CLASS** | **FRAMES**    **NO FRAMES**     **All Classes** |  |
| SUMMARY: NESTED \| FIELD \| CONSTR \| METHOD | DETAIL: FIELD \| CONSTR \| METHOD |  |


---
